# Chlorogenic Acid-Loaded Mesoporous Silica Nanoparticles Modified with Hexa-Histidine Peptides Reduce Skin Allergies by Capturing Nickel

**DOI:** 10.3390/molecules27041430

**Published:** 2022-02-21

**Authors:** Tianyu Wang, Liying Yin, Zheng Ma, Yanrong Zhang

**Affiliations:** 1 College of Food Science and Engineering, Jilin Agriculture University, Changchun 130118, China; wangtianyuccdx@126.com; 2 College of Food Science and Engineering, Changchun University, Changchun 130012, China; yly202201@126.com; 3 Department of Thoracic Surgery, Qilu Hospital, Cheeloo College of Medicine, Shandong University, Jinan 250012, China

**Keywords:** nickel, chlorogenic acid, absorption, allergic contact dermatitis, MSN

## Abstract

Nickel-induced contact dermatitis is a severe allergic reaction to objects or environments that contain nickel. Many nanomaterials have been developed to reduce skin allergies by capturing nickel, but few agents are effective and safe. In this work, mesoporous silica nanoparticles (MSN) were synthesized and decorated with hexa-histidine peptides (denoted as MSN-His^6^), making it a strong nickel chelator. Subsequently, a dietary polyphenol, chlorogenic acid, was loaded into the mesopores of MSN (denoted as MSN-His^6^@CGA), realizing the potential of its anti-inflammatory properties. In vitro and in vivo experiments revealed that the synthesized MSN-His^6^@CGA nanoparticles exhibited more stable and stronger chelation, better biocompatibility, and ideal allergy-relieving ability, whether for environmental metal contamination or for allergic contact dermatitis caused by prolonged nickel exposure. Thus, the application of mesoporous silica-based nanoparticles may represent an ideal approach to alleviate skin allergies by capturing nickel, which would benefit people who suffer from metal-induced contact dermatitis.

## 1. Introduction

Allergic contact dermatitis (ACD) is the most common disease in dermatology. There are three types of contact dermatitis, including irritant contact dermatitis, ACD, and photocontact dermatitis in clinic [1]. Among them, ACD was traditionally considered as an immune-mediated disease driven by Th2 cells, but suppressed Th1 cells mediate cellular immunity and cause ACD. ACD is problematic. It is the most widely spread dermatitis across the world, and is constantly increasing, since ACD is an occupation- and environment-related disease, caused by metal contact allergens, such as nickel, chromium, lead, and cobalt [2]. For example, nickel ions are solubilized from nickel-containing cosmetics or jewelry by sweat, and subsequently serve as allergens that percutaneously invade the stratum corneum barrier, which is the water-impermeable outer layer of the skin. Notably, ACD is a type IV delayed hypersensitivity reaction that typically develops after repeated or prolonged topical exposure to metal allergens. Once metal ions come into contact with pattern-recognition receptors (PRRs), an inappropriate immune response is initiated, leading to the antigen-presenting function of resident dendritic cells and Langerhans cells, the generation of allergen-specific T-cells, and lymphocyte activation. This initial sensitization phase is followed by the production of pro-inflammatory mediators, including interleukin-1β (IL-1β) and interferon-γ (IFN-γ) [3]. T-cell receptors (TCR) interacting with mimotope p7 lysine mimics nickel ions and causes a T-cell-mediated allergy to nickel [4]. Consequently, metal ions can trigger the innate immunologic response and activate T cells, eventually resulting in the development of the inflammatory cytokine release that is seen in patients with ACD [1,5,6].

Some heavy metals are essential for plants and animals when in low concentrations [7]. In fact, even though no nickel-containing enzyme or cofactor can be found in higher animals, some research showed that enzyme activities declined because the catalytic center consisted of metal ions when nickel depletion occurred [2]. On the contrary, high nickel and lead exposure drive many diseases, including carcinogenesis and allergies. To the human skin, high metal exposure can result in ACD. An ideal preventive treatment for nickel-induced contact dermatitis is a skin toner that can be applied to effectively capture nickel or two other valence metal ions in a short time, with high absorption, low toxicity, and immunogenicity. Inorganic materials, such as porous silica nanoparticles, have attracted our attention, limited not only in the chemistry industry, but also in healthcare, medical applications, and cosmetics [8,9,10,11]. Mesoporous silica nanoparticle (MSN)-based nanostructures have been widely used as carriers for bioactive compounds due to their excellent biocompatibility, high surface areas, tunable pore sizes, large pore volumes, and rich morphology [12,13]. As the “generally recognized as safe” (GRAS) agents of the United States Food and Drug Administration, calcium carbonate (CaCO_3_) and calcium phosphate (CaPO_4_) nanoparticles have been applied to reduce skin irritation [5,14], whereas the development of mesoporous silica nanoparticles, which present excellent biosafety and biocompatibility, are limited in use for allergic skin reactions to heavy metal ions. Adsorption dependence on smaller grain sizes of zeolite and higher pH values of solutions lead to an increase in adsorption capacity [15].

The hexa-histidine peptides (His^6^) are used in immobilized metal ion affinity chromatography, where N- or C-terminal recombinant proteins have consecutive polyhistidine fragment repeats that can be purified from a mixture of proteins by using a nitrilotriacetic acid (NTA)/Ni^2+^ column with high affinity and selectivity [16,17]. As is known, the stable octahedral structures around Ni^2+^ are formatted octahedral structures. Expanding this conjugation to the absorption of heavy metal ions between MSN coupling His^6^ tag and Ni^2+^-rich aqueous solution, it would be feasible to remove and recycle heavy metal ions. Furthermore, severe air and water pollution seriously affect human health, and rationally designed nanomaterial systems could efficiently scavenge heavy metal pollution and associated life-threatening diseases.

**Figure 1:**
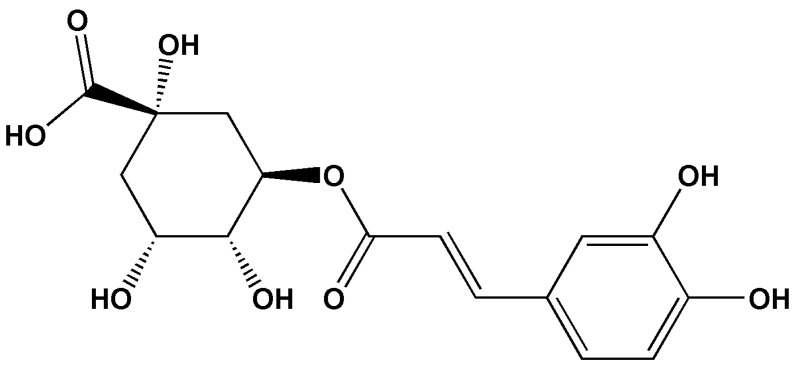
The chemical structure of chlorogenic acid (C_16_H_18_O_9_).

Flavonoids and phenolic acids have structural similarities and both belong to the polyphenol family, which are secondary metabolites widely produced in plants. Chemically, the structures of phenolic compounds possess one or more aromatic rings with hydroxyl groups attached [18]. The poor physicochemical characteristics of these, such as insolubility and thermo-optical instability, restrict their application in medicine and the food industry. In recent years, flavonoids such as 2′,4′-dihydroxychalcone have been obtained from herbs and identified as natural biologically active compounds [19]. Chlorogenic acid (CGA, 5-CQA, Figure 1) is an important dietary phenolic acid that accumulates in substantial levels in plants, such as cherry, apple, grape, pear, plum, carrot, tea and coffee. CGA exerts many biological and pharmacological effects which have drawn substantial attention in the development of food additives and dietary supplements. CGA was found to perform antioxidation and anti-inflammation by inhibiting the release of cytokines and inactivating MAPKs and the NF-κB pathway [20,21,22]. CGA also has bactericidal effects, as it is resistant to harmful bacteria and inhibits multidrug efflux systems of drug-resistant bacteria [23]. Furthermore, CGA showed the potential to antagonize lead-induced nephrotoxicity and hepatotoxicity [24]. Mesostructured silica-based adsorbents functionalized with amino groups have been used in the separation of CGA, indicating that CGA can be absorbed by MSN [25]. To increase the valorization, enhance the stability and reduce the sensitivity of polyphenols, Brezoiu et al. reported grape pomace polyphenolic extracts being encapsulated into MSN, thus resulting in a radical scavenger effect [26]. Wang et al. present the antibacterial activity of CGA-loaded MSN through the accumulation of reactive oxygen species [27].

In this study, MSN decorated with hexa-histidine peptides, making for an efficient and strong chelator of two-valence heavy metal ions. CGA were subsequently encapsulated into MSN-His^6^ to exert antibacterial, anti-oxidative and anti-inflammatory activities. Our results showed that hexa-histidine peptide-functionalized MSN exhibited a strong absorption capacity, better biocompatibility and more stable chelation, whether for environmental metal contamination or for allergic contact dermatitis caused by prolonged metal ion exposure, and the ideal MSN-His^6^ adsorbent loaded with a natural compound leading to the effect of interest has potential application prospects.

## 2. Results

### 2.1. Synthesis and Characterization of MSN-His^6^

**Scheme 1:**
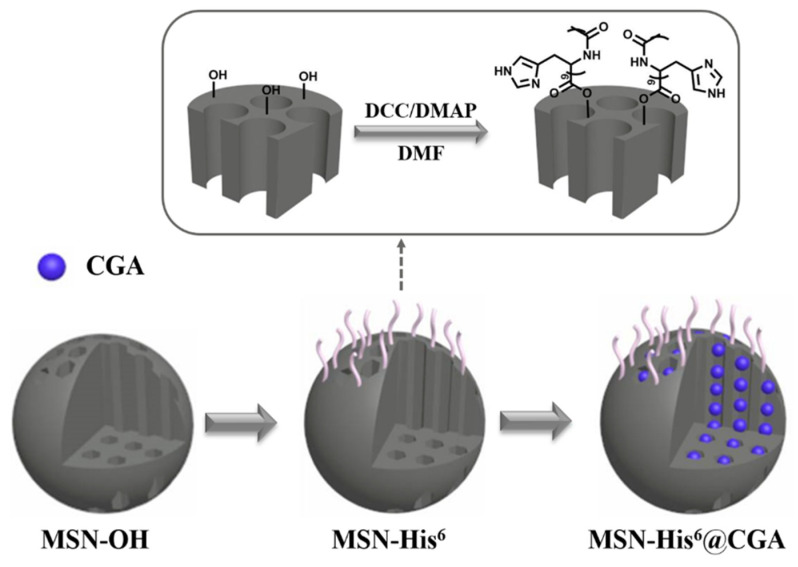
Preparation of MSN-His^6^@CGA composites.

**Figure 2:**
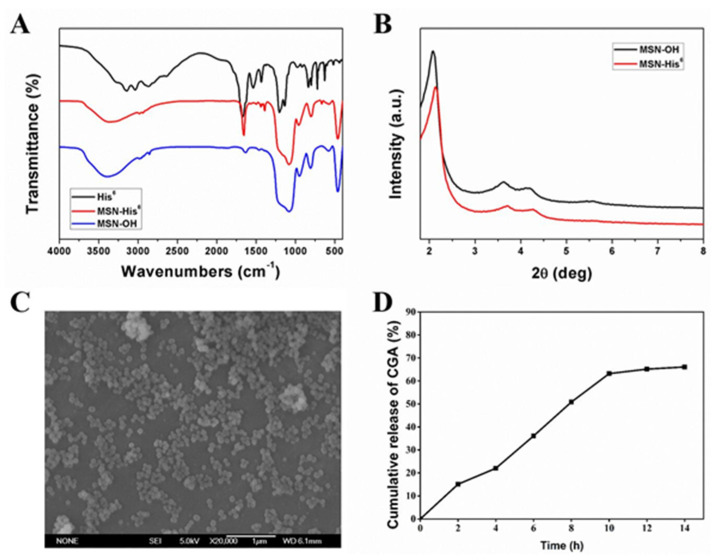
Characteristics of MSN-His^6^. (**A**) FT-IR spectra; (**B**) powder XRD patterns; (**C**) the SEM images of MSN-His^6^; (**D**) CGA release curve of MSN-His^6^@CGA in PBS.

Histidine is an amino acid having a heterocyclic ring and imidazole group, with many extranuclear electrons accumulating around this group. The structure of histidine attracts positively charged substance including transition metal elements, such as nickel, copper, zinc, lead, and cobalt. This principle has been widely used in protein purification. The His^6^ fusion tags are usually engineered into target proteins, so then the interest protein can be purified with a nitrilotriacetic acid (NTA)/Ni^2+^ column with high affinity and selectivity by an affinity chromatography technique [16,17]. According to the performance of His^6^ tag, the peptides of His^6^ were decorated onto MSNs. The synthesis procedure for the MSN-His^6^ composite is illustrated in Scheme 1. Briefly, MSN-OH was prepared first using a base-catalyzed sol-gel method. The peptides of His^6^ were then conjugated to the surface of MSN-OH in the presence of a catalyst. To further improve the anti-inflammatory activities, the CGA was loaded into the mesopores of the MSN-His^6^ composite. As shown in Figure 2, the synthetic process of MSN-His^6^ was confirmed by FT-IR analysis and powder X-ray diffraction (XRD). The presence of peptide on the MSN is verified by the characteristic absorption peaks at 2800–3000 cm^−1^ and 1420–1700 cm^−1^, in the FT-IR spectrum of MSN-His^6^, which can be assigned to the vibrations of CH_3_ and CH_2_, as well as the C=O and N-H of peptides. The FT-IR results demonstrated that peptides of His^6^ were successfully decorated on the surface of MSN-OH. The XRD patterns of the synthesized MSN and MSN-His^6^ nanoparticles exhibited characteristic diffraction peaks of MCM-41 (100, 110 and 200), corresponding to 2θ = 2.202, 2θ = 3.737 and 2θ = 4.298 respectively. The FT-IR and XRD data showed electrostatic interactions between NH_2_-His^6^ and Si-OH of MSN. The morphology of the MSN-His^6^ nanoparticles was evaluated by SEM. The SEM images in Figure 2C show uniform spherical morphology with a diameter of 120 nm. MSN-His^6^@CGA was dispersed in PBS (pH 7.4) containing 0.1% Tween 80 at room temperature, the supernatant containing released CGA was collected and the amount of CGA was determined by dialysis and UV spectra. The results showed that the CGA loading capacity was about 15 wt% and the cumulative release of CGA from MSN-His^6^@CGA nanoparticles at 10 h was 63.22%, maintaining stability (Figure 2D). To further assess the CHN elemental composition of MSN-His^6^ nanoparticles, CHN elemental analyses were conducted. As shown in Table 1, the CHN elemental composition of MSN-His^6^ was higher than that of MSN-OH nanoparticles, demonstrating that the peptides of His^6^ were successfully decorated onto MSNs.

### 2.2. Effect of pH Value and Adsorption Time of MSN-His^6^ on Capacity to Bind Nickel

**Figure 3:**
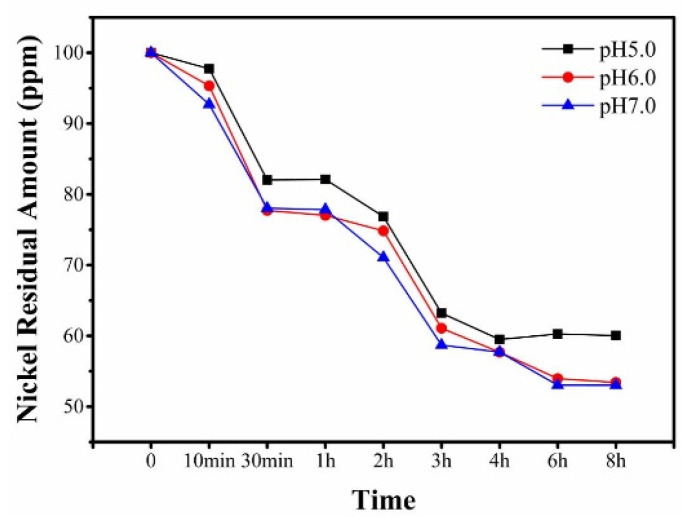
Effect of solution initial pH (5.0, 6.0 and 7.0) on the sorption of nickel at different adsorption times (0–8 h).

The solution pH and adsorption time may affect the sorption of nickel. Thus, the nickel removal performance of the MSN-His^6^ was further investigated. As shown in Figure 3, the curves of residual amount indicated that the adsorption of nickel was slightly increased by increasing the initial pH of the solution at different adsorption times. The adsorption of nickel stayed constant when the adsorption time was around 4 h at different pH values. The solution with a pH within neutral range does not affect the sorption of nickel significantly. Hence, the chelation of MSN-His^6^ is stable.

### 2.3. Cytotoxicity of Nanoparticles

**Figure 4:**
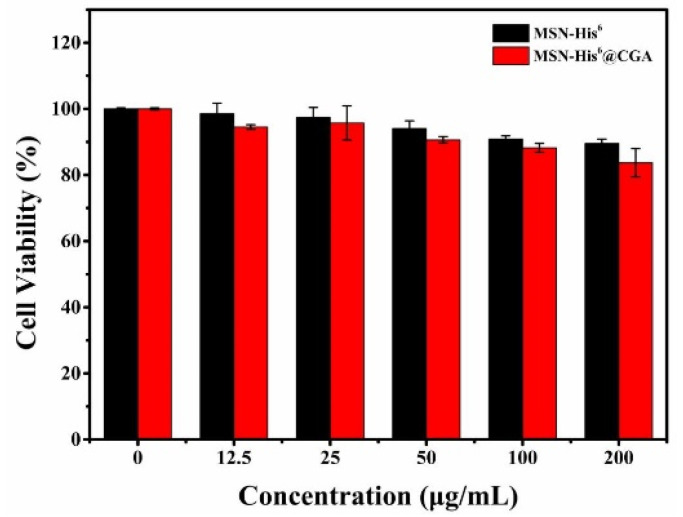
Cell viability of BJ cells after incubation with MSN-His^6^ (12.5, 25, 50, 100, 200 μg/mL) or MSN-His^6^@CGA (12.5, 25, 50, 100, 200 μg/mL) for 12 h.

The protein corona mitigates cytotoxicity and influences nanoparticle pathophysiology of the mesoporous silica nanostructures [28]. BJ cells were incubated with different concentrations of MSN-His^6^ (12.5, 25, 50, 100, 200 μg/mL) or MSN-His^6^@CGA (12.5, 25, 50, 100, 200 ug/mL) solution for 12 h. As shown in Figure 4, no obvious cell cytotoxicity of the MSN-His^6^ or MSN-His^6^@CGA treatment was observed even when the concentration of nanoparticles was as high as 200 μg/mL. The results demonstrated that the introduction of hexa-histidine peptides could improve the biocompatibility of MSN and the incorporation of CGA will cause toxicity to normal cells.

### 2.4. Nickel Removal and Anti-Inflammatory Potential of Nanoparticles

**Figure 5:**
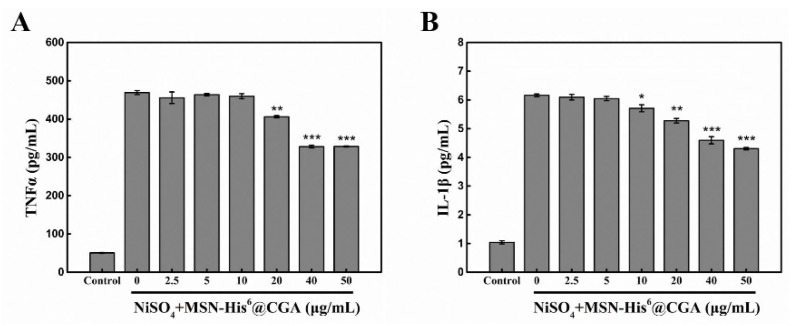
Effect of MSN-His^6^@CGA on cytokine production in nickel-stimulated Raw 264.7 cells. Cells were treated with MSN-His^6^@CGA (0, 2.5, 5, 10, 20, 40 and 50 μg/mL) and NiSO_4_·6H_2_O (0.05 M, 1.3% (wt/vol) NiSO_4_·6H_2_O) for 12 h. Secretion levels of TNFα and IL-1β were detected by ELISA. Error bars represent standard deviation. Statistical analysis was determined using two-tailed *t*-test. The results were considered significant as * *p* < 0.05, ** *p* < 0.01, and *** *p* < 0.001 compared with the independent NiSO_4_ treatment. The amounts of TNFα and IL-1β in MSN-His6@CGA-treated (50 μg/mL) Raw 264.7 cells were 328.50 (**A**) and 4.30 pg/mL (**B**).

To further to understand the anti-inflammatory effect of nanoparticles, a mouse macrophage cell line, Raw 264.7 cells, were used in the test of anti-inflammatory activity. In brief, cells were cultured in a 96-well plate and pre-treated with nickel in the presence or absence of CGA-loaded MSN-His^6^ nanoparticles for 4 h; the cells were later incubated for a total 24 h. Subsequently, the production of IL-1β and TNFα was measured by an ELISA kit. The results showed that the release of both IL-1β and TNFα increased in cell culture supernatant after being sensitized with nickel (Figure 5A,B, 0 μg/mL), and then decreased with the incubation of MSN-His^6^@CGA (2.5, 5, 10, 20, 40 and 50 μg/mL). The amounts of TNFα and IL-1β in MSN-His^6^@CGA-treated (50 μg/mL) Raw 264.7 cells were 328.50 (A) and 4.30 pg/mL (B), which decreased significantly by nanoparticle treatments, confirming the anti-inflammation property of CGA-loaded MSN-His^6^ nanoparticles in vitro.

**Figure 6:**
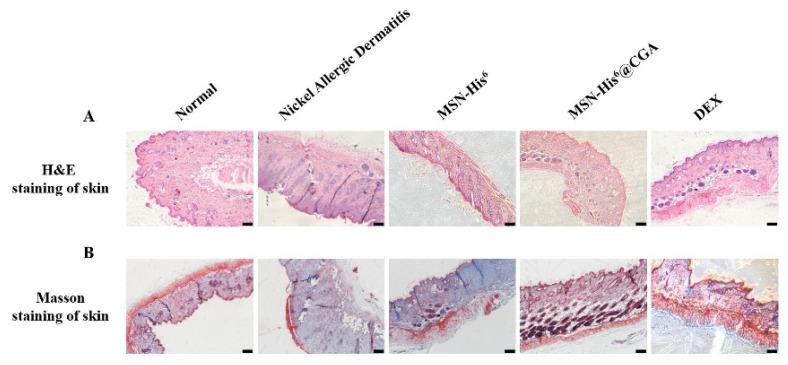
Histopathological analysis of mice skin by using (**A**) haemaroxylin-eosin (H&E) staining and (**B**) Masson’s trichrome staining. Images shown are representatives from skin tissues taken from saline (normal), nickel (nickel-allergic dermatitis), MSN-His^6^, MSN-His^6^@CGA and DEX treatment. Scale bar is 100 μm.

**Figure 7:**
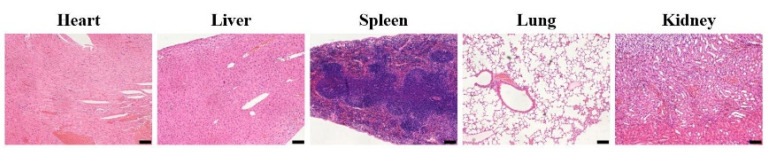
Histopathological analysis of mice main organs after treated with MSN-His^6^@CGA. Scale bar is 100 μm.

To investigate the nickel removal performance of nanoparticles, BALB/c mice were challenged with a topical application of NiSO_4_ at the indicated time points, followed by challenges thereafter. Once nickel-allergic dermatitis was induced, mice were topically applied with solution of MSN-His^6^ or MSN-His^6^@CGA (50 μg/mL) daily for 2 weeks. In vivo experiments were performed in collaboration with mice models with erythema, which were exposed to large doses of nickel in the presence or absence of the nanoparticles. The skin specimens were subjected to H&E staining or Masson’s trichrome staining. As shown in Figure 6, as a blank control, the saline treatment was applied to normal mice and the results of H&E and Masson’s staining did not exhibit obvious skin injury. The nickel-sensitized mice without treatment were set as the negative control. As the figure shows, skin of nickel-allergic mice showed obvious damage or abnormalities. ACD-like lesions were induced after exposures to nickel. Meanwhile, in this work, the synthesized nanoparticles were utilized to treat nickel-allergic dermatitis on the dorsal skin of mice. The results revealed that the impaired skin of the nickel-allergic mice was ameliorated with an application of MSN-His^6^, MSN-His^6^@CGA. Especially for MSN-His^6^@CGA treatment, images show a better alleviating effect on skin allergy and dermatitis response, such as skin scaling, exfoliation and edema. As expected, the nickel-allergic mice that were subjected to a positive-drug (DEX) parallel treatment exhibited significantly repaired features. Importantly, there was no obvious histological changes in nickel-allergic main organs with the treatment of MSN-His^6^@CGA (Figure 7), indicating that MSN-His^6^@CGA has good biocompatibility.

## 3. Discussion

Metal ions such as contact allergens are common in cosmetics, jewelry, personal care products and modern industrial products, making allergic contact dermatitis the most common disease in dermatology. The history of chemical antigens serving as skin sensitizers can be traced back to the early 1930s [29]. In this work, we synthesized a safe functionalized MSN to ensure the chelation of heavy metal ions and anti-inflammatory activities.

The simple synthetic method of MSN takes cationic surfactants as templates [30], and has been widely applied in the preparation of porous nanoparticles with high surface areas, appropriate pore sizes, large pore volumes and good dispersity. The functionalized MSN possesses the ability of a controlled bioactive biomolecule for therapeutic release. Some porous silicon patents for drug delivery have been granted, primarily for implants, as well as ophthalmologic and immunotherapy applications [31]. In addition, the application of porous materials as nanosorbents has found an emerging role in solving environmental problems such as heavy metals contamination due to their unique physical and chemical properties [32,33,34]. In previous studies, biomolecular corona and the opsonization of complement factors on nanoparticles change the design’s original intention [35,36]. However, the small-sized peptides have a simple structure, making them more stable, with lower immunogenicity, and they can more easily penetrate cells than other compounds or antibodies [37]. Therefore, the hexa-histidine peptides were not only employed as chelators of nickel based on their strong affinity to two-valence heavy metal ions, but also as cell-penetrating peptides according to the positively charged histidine.

As is well known, allergens such as nickel can be recognized by the receptors which are expressed on antigen-presenting cells, such as Langerhans cells and dermal DCs, and next will be captured by conventional CD^8+^ and CD^4+^ T cells [1,38]. Obviously, nickel can trigger delayed-type hypersensitivity (DTH) by helper T (Th) cells of “signal calling”. Thereafter, the non-specific part of DTH involves the macrophages and neutrophils that are recruited and activated by cytokines, such as IL-2 and IFN-γ [39]. Bioactive food compounds such as chlorogenic acid have been used as nutraceuticals, mainly due to their antioxidant and anti-inflammatory activities [22]. Given the aforementioned excellent performances of MSN, they are not only of increasing importance for use in adsorption, but have also emerged as a promising delivery system in recent studies. Thus, to further improve anti-inflammatory ability, CGA was loaded into the mesopores of MSN in view of the excellent anti-inflammation activities. The rapid inflammatory response is produced in nickel-stimulated Raw 264.7 macrophages, which release pro-inflammatory cytokines, such as IL-1β and TNFα. This leads to the subsequent recruitment of large numbers of sensitized immune cells. As expected, the ELISA results indicated that CGA-loaded MSN-His^6^ nanoparticles can effectively prevent the release of IL-1β and TNFα from nickel-stimulated Raw 264.7 macrophages and may therefore reduce nickel-induced skin allergy. We then investigated the in vivo effect of CGA-loaded MSN-His^6^, and it is more appropriate to evaluate the nickel removal performance of nanoparticles. In the application of treatments to the broken skin of nickel-allergic mice, the presence of CGA-loaded MSN-His^6^ or dexamethasone reduced skin exposure to nickel ions, and the resulting alleviating effect on skin allergy and dermatitis response.

In conclusion, we have shown that MSN-His^6^ nanocomposites can efficiently capture nickel ions, and the introduction of histidine tag is improving efficiency of nickel removal and enhancing biocompatibility of MSN. A natural compound, chlorogenic acid, was loaded into MSN-His^6^ nanocomposites, to alleviate the symptoms of inflammation in nickel-allergic mice. Both in vitro and in vivo results in this study revealed that chlorogenic acid-loaded mesoporous silica nanoparticles modified with hexa-histidine peptides nanoparticles could bind to nickel, which might be beneficial to alleviate nickel-induced contact dermatitis.

## 4. Materials and Methods

### 4.1. Materials

Cell culture medium (MEM), fetal bovine serum (FBS), penicillin-streptomycin solution and trypsin-ethylenediaminetetraacetic acid (EDTA) were purchased from Gibco (Grand Island, NY, USA). The modified Masson’s trichrome stain kit and hematoxylin-eosin (H&E) were obtained from Solarbio Science & Technology Co., Ltd. (Beijing, China). 3-(4,5-Dimethylthiazol-2-yl)-2,5-diphenyltetrazolium bromide (MTT, 98%), dexamethasone (DEX, ≥98%), and 1-chloro-2,4-dinitrobenzene (DNCB, 97%) were purchased from Sigma-Aldrich (St. Louis, MO, USA). Cetyltrimethylammonium bromide (CTAB), sodium hydroxide (NaOH), tetraethylorthosilicate (TEOS), dicyclohexylcarbodiimide (DCC), dimethyl formamide (DMF), nickel sulfate hexahydrate (NiSO_4_·6H_2_O) and dimethylaminopyridine (DMAP) were purchased from Sinopharm Chemical Reagent Co., Ltd. (Beijing, China). Chlorogenic acid (CGA, CAS: 327-97-9, purity ≥ 98%) was purchased from Shanghai Yuanye Bio-Technology Co., Ltd. (Shanghai, China).

### 4.2. Synthesis of Peptides

The hexa-histidine polypeptide was purchased from GL Biochem Ltd. (Shanghai, China). Simply, the amino terminal modified polypeptides (NH_2_-His^6^, short denote as His^6^) were synthesized by the solid-phase method using Fmoc chemistry, and crude products were purified by reversed-phase column chromatography (RP-HPLC) and validated by mass-spectrography. Peptide purification was 98%.

### 4.3. Synthesis and Characterization of MSN-His^6^@CGA

MSN were synthesized according to previous studies [40,41]. In total, 0.1 g of CTAB and 0.35 mL of NaOH (2 M) were dissolved in 48 mL of deionized water under 80 °C for 30 min. Then 0.5 mL of TEOS was added into the mixture with vigorously stirring at 80 °C for 3 h. The product was collected then washed with deionized water and absolute methanol 5 times. After centrifugation and freeze-drying, the prepared MSN-OH was obtained and stored at room temperature. The direct esterification was carried out in DMF by use of DCC/DMAP as catalyst in the conjugation of NH_2_-His^6^ peptide and MSN-OH. In brief, polypeptide fragments (NH_2_-His^6^, 1 mg), MSN (20 mg), DMAP (1 mg) and DCC (1 mg) were dissolved in anhydrous DMF (5 mL) solution. We then gently agitated the mixture at 25 °C for 5 days and the products were gained by vacuum distillation from the reaction liquid. To analyze the characterization of MSN-His^6^, the infrared (IR) spectra and thermo gravimetric analyzer (TGA) were used, and the samples dispersed in KBr pellets were performed on a Perkin-Elmer spectrum 430 FT-IR spectrometer. CHN elemental analysis of MSN-His^6^ nanoparticles was conducted with a micro elemental analyzer. Finally, the MSN-His^6^ composite (10 mg) was dispersed in an ethanol solution of CGA (1 mg/mL) through ultrasound. The mixture was stirred for 24 h at room temperature. After centrifugation and washing, the MSN-His^6^@CGA was collected and dried in a freezer dryer. The drug loading and release rate were calculated. In brief, CGA loading rate = weight of MSN-His^6^@CGA/MSN-His^6^. Then, dialysis was performed to measure the release of CGA. In short, 1 mg/mL of CGA/ MSN-His^6^ was added to a dialysis bag with a molecular weight cutoff of 10 kD. The dialysis bag was immersed in PBS containing 0.1% Tween 80 for 14 h and the release solution was collected every 2 h and replaced with the fresh PBS. The release of CGA was measured by an ultraviolet spectrophotometer at 320 nm. In addition, the morphologies of particles were observed using a scanning electron microscope.

### 4.4. Detection of Absorption Capacity of MSN-His^6^

To evaluate the removal of nickel ions, MSN-His^6^ powder (solid/liquid ratio of 10 g/L) was dispersed in 5 mL NiSO_4_·6H_2_O aqueous solution at a concentration of 100 mg/L. Dilute aqueous solution of HCl or NaOH was added to adjust pH values to 6.5, 7.0 and 7.5. The adsorption time was 0–8 h at 25 °C. Afterwards, the mixture was stirred continuously and the residual amount of nickel in the supernatant was quantified using Thermo Scientific iCAP 7600 DUO ICP-OES (Thermo Fisher Scientific, Waltham, MA, USA).

### 4.5. Cytotoxicity of Nanocomposites

The MTT assay was performed to evaluate the cytotoxicity of nanoparticles. Human fibroblast BJ cells were cultured in Eagle’s minimum essential medium (EMDM) containing 10% fetal bovine serum, in 37 °C and 5% CO_2_ atmosphere. Cells were cultured for three passages. In the subculturing procedure, the cell layer was rinsed with 0.25% (*w*/*v*) Trypsin-0.53 mM EDTA solution. Cells were seeded in 6-well plates at a density of 4 × 10^3^/well for 12 h and were pre-treated with or without nickel ions (0.05 M, 1.3% (wt/vol) NiSO_4_), MSN-His^6^ (12.5, 25, 50, 100, 200 µg/mL) or MSN-His^6^@CGA (12.5, 25, 50, 100, 200 µg/mL) solution, then incubated in 96-well plates for another 12 h. Following washes and the addition of the MTT containing fresh medium, absorbance at 490 nm was measured using a standard plate reader.

### 4.6. ELISA Assays

Raw 264.7 macrophages were seeded in 12-well plates (5 × 10^5^ cells per well) and incubated at 37 °C and 5% CO_2_ atmosphere for 12 h. The cells were co-treated with NiSO_4_·6H_2_O (0.05 M, 1.3% (wt/vol) and MSN-His^6^@CGA (0, 2.5, 5, 10, 20, 40 and 50 μg/mL) for 12 h. The supernatant were harvested and the secretion levels of TNFα and IL-1β were detected by ELISA according to the manufacturer’s instructions.

### 4.7. Mice Treatments

BALB/c mice were purchased from Beijing Vital River Laboratory Animal Technology Co., Ltd. (Beijing, China). The animal experiment was approved by the Laboratory Animal Ethical and Welfare Committee of Shandong University Cheeloo College of Medicine. Approved No is KYLL-2021(KS)-733. This study does not involve any human or patients testing. All in vivo experiments were performed with 8-week-old mice (20–22 g, female), after which they were allowed to acclimate for 1 week in a pathogen-free barrier system. The mice were shaved to remove dorsal hair in an area of 3 × 3 cm^2^. The nickel-allergy model was established by sensitization with an aqueous 20% solution of NiSO_4_·6H_2_O as previously described [14]. In brief, the dorsal skin of mice was smeared with 50 μL NiSO_4_·6H_2_O solution on day 1 to 7; on day 14, sensitized mice were challenged topically by smearing 20 μL of 0.4% NiSO_4_·6H_2_O solution every 3 days. Saline was used as a control. Nickel-allergy symptoms were recorded blind and assessed after challenge, referring to the method of establishing of allergic contact dermatitis [38,42]. Mice were randomly assigned to different experimental groups (5 mice each). 21-days later, following the application of the solution of MSN-His^6^ or MSN-His^6^@CGA (50 μg/mL). Dexamethasone treatment (DEX) was used as a positive control. The solution for different treatments was topically performed to the dorsal skin of nickel-sensitized mice every day for 2 weeks.

### 4.8. Histological Analysis

Skin biopsies and organ dissections were taken from the CO_2_-asphyxiated sacrificed mice and were fixed in 10% of neutral buffered formalin. All samples were embedded in paraffin wax, sectioned at 6 μm, were stained on clean adhesion microscope slides with haemaroxylin-eosin (HE) and modified Masson’s trichrome stains in accordance with the manufacturer’s protocols. The images of the stained sections were captured with a fluorescent Olympus DP72 microscope (microscope, Tokyo, Japan).

### 4.9. Statistical Analysis

Data were expressed as mean ± SD. The paired Student’s *t* test was applied to compare the two groups involved in the experiments. The results were considered statistically significant at * *p* < 0.05, ** *p* < 0.01, and *** *p* < 0.001 compared with the control group.

## Figures and Tables

**Table 1:** Element contents of materials.

	N%	C%	H%
MSN-OH	0.22	4.62	1.73
MSN-His^6^	3.10	9.01	2.123

## Data Availability

Not applicable.

## Sample Availability

Samples of the compounds are available from the authors.

## Abbreviations

ACD	allergic contact dermatitis
MSN	mesoporous silica nanoparticles
Th cells	T helper cells
TCR	T-cell receptor
IL-1β	interleukin-1β
IFN-γ	interferon-γ
His^6^	the hexa-histidine peptides tag
CGA	chlorogenic acid
MSN-His^6^	MSN coupling His^6^ tag
MSN-His^6^@CGA	CGA-loaded MSN-His^6^
DEX	dexamethasone
